# Carbonic anhydrase IX inhibitor S4 triggers release of DAMPs related to immunogenic cell death in glioma cells via endoplasmic reticulum stress pathway

**DOI:** 10.1186/s12964-023-01180-7

**Published:** 2023-06-29

**Authors:** Jing Cui, Huizhe Xu, Ji Shi, Kun Fang, Jia Liu, Feng Liu, Yi Chen, Haiyang Liang, Ye Zhang, Haozhe Piao

**Affiliations:** 1 Department of Neurosurgery, Cancer Hospital of China Medical University, Cancer Hospital of Dalian University of Technology, Liaoning Cancer Hospital & Institute, No.44 Xiaoheyan Road, Dadong District, Shenyang, 110042 China; 2 Central Laboratory, Cancer Hospital of China Medical University, Cancer Hospital of Dalian University of Technology, Liaoning Cancer Hospital & Institute, No.44 Xiaoheyan Road, Dadong District, Shenyang, 110042 China; 3 Institute of Cancer Stem Cell, Dalian Medical University, No.9 Lvshun South Road, Lvshunkou District, Dalian, 116044 China

**Keywords:** Carbonic anhydrase IX inhibitor, Immunogenic cell death, Glioma, Endoplasmic reticulum (ER) stress, S4

## Abstract

**Background:**

Immunogenic cell death (ICD), which releases danger-associated molecular patterns (DAMP) that induce potent anticancer immune response, has emerged as a key component of therapy-induced anti-tumor immunity. The aim of this work was to analyze whether the carbonic anhydrase IX inhibitor S4 can elicit ICD in glioma cells.

**Methods:**

The effects of S4 on glioma cell growth were evaluated using the CCK-8, clonogenic and sphere assays. Glioma cell apoptosis was determined by flow cytometry. Surface-exposed calreticulin (CRT) was inspected by confocal imaging. The supernatants of S4-treated cells were concentrated for the determination of HMGB1and HSP70/90 expression by immunoblotting. RNA-seq was performed to compare gene expression profiles between S4-treated and control cells. Pharmacological inhibition of apoptosis, autophagy, necroptosis and endoplasmic reticulum (ER) stress was achieved by inhibitors. In vivo effects of S4 were evaluated in glioma xenografts. Immunohistochemistry (IHC) was performed to stain Ki67 and CRT.

**Results:**

S4 significantly decreased the viability of glioma cells and induced apoptosis and autophagy. Moreover, S4 triggered CRT exposure and the release of HMGB1 and HSP70/90. Inhibition of either apoptosis or autophagy significantly reversed S4-induced release of DAMP molecules. RNA-seq analysis indicated that the ER stress pathway was deregulated upon exposure to S4. Both PERK-eIF2α and IRE1α- XBP1 axes were activated in S4-treated cells. Furthermore, pharmacological inhibition of PERK significantly suppressed S4-triggered ICD markers and autophagy. In glioma xenografts, S4 significantly reduced tumor growth.

**Conclusions:**

Altogether, these findings suggest S4 as a novel ICD inducer in glioma and might have implications for S4-based immunotherapy.

Video Abstract

**Supplementary Information:**

The online version contains supplementary material available at 10.1186/s12964-023-01180-7.

## Introduction

Gliomas are the most common type of primary intracranial tumors. Conventional therapies, including surgery, radiotherapy, and pharmacotherapy (typically chemotherapy with temozolomide), have improved the median survival to some extent, the prognosis for patients with malignant glioma, especially glioblastoma remains poor [1]. Accumulating evidence reveals that some conventional treatments, in addition to their direct cytotoxic effect, could induce an antitumor immune response [2]. Immunogenic cell death (ICD), named after the immunogenicity of dying/dead cancer cells, is induced by certain types of therapies [3, 4]. ICD has emerged as a key component of therapy-induced anti-tumor immunity [5]. Of note, some ICD inducers can function in synergy with other types of immunotherapy, such as immune checkpoint inhibitors therapy to potentiate their effectiveness [6]. ICD is characterized by the emission of immuno-stimulatory molecules, including damage-associated molecular patterns (DAMPs) such as cell surface exposure of the endoplasmic reticulum protein calreticulin, secretion of ATP, and release of the chromatin-binding protein HMGB1 [3, 7]. Other DAMPs such as heat-shock proteins (HSP90 and HSP70) are also exposed on the outer membrane of the dying cells or released [8–10]. These DAMP molecules play a key role in activating dendritic cells (DCs) to engulf dying tumor cells, to process and present released tumor antigens to T cells [11–13]. Given the therapeutic potential of ICD in several types of cancer, a few pre-clinical investigations have shown that malignant gliomas might benefit from ICD-based therapies [4].

Carbonic anhydrase IX (CAIX), a tumor-associated, cell-surface glycoprotein expressed in response to hypoxia, plays a pivotal role in pH homeostasis, which is essential for tumor cell survival. Literature documents that CAIX is implicated in cancer progression [14–16], and has been validated as a promising new anticancer target [17]. Several CAIX inhibitors such as SLC-0111, have been developed and shown to be effective in reducing primary tumor growth in vitro and in vivo [18]. S4, a CAIX specific sulfamate inhibitor [19], exhibited anti-proliferative efficacy in breast and colorectal tumor cells [20–23]. In addition, an antimetastatic effect of S4 in breast carcinoma xenografts was reported [19]. Of interest, S4 has shown to potentiate the efficacy of standard treatment modalities, such as doxorubicin in breast cancer [24], cisplatin in small cell lung cancer [25], suggesting a S4-based combination strategy against tumors. However, whether S4 would suppress glioma growth has not been investigated.

In the present study, we examined the effect of treatment with S4 on glioma cell growth in cell cultural systems and in murine models of glioma. Our data demonstrate that S4 triggers ICD in glioma cells via the induction of ER stress pathway. These preliminary data could represent the basis for further studies to explore a potential role of S4 as an ICD inducer for treatment of malignant glioma.

## Materials and methods

### Cell lines, regents and antibodies

Human oligodendroglioma (low grade glioma) cell line Hs683, glioma cell lines, LN229 and U87MG and human acute monocytic leukemia cell line THP-1 were obtained from the American Type Culture Collection (ATCC). The human astrocytes HA cell line was obtained from ScienCell. Hs683 and HA were cultured with DMEM which contained 10% FBS. LN229 were cultured with 5% FBS DMEM. U87MG was cultured with 10% FBS MEM EAGLE. THP-1 was cultured with RPMI-1640 which contained 10% FBS and 0.05 mM 2-mercaptoethanol. All cells were maintained at 37 °C in a humidified incubator with 5% CO_2_. For 3D culture, cells were seeded in ultra low attachment culture plates with DMEM/F12 (FBS deprived) medium supplied with basic fibroblast growth factor (bFGF) 10 ng/ml, epidermal growth factor (EGF) 20 ng/ml and 1 × B27.

S4 was obtained from Tocris Bioscience. Mitoxantraone (MTX), Necrostain-1, Z-VAD-FMK, 4μ8C were purchased from SELLECK. GSK2606414 was purchased from Apexbio, ISRIB was purchased from MCE. Chloroquine (CQ) and phorbol 12-myristate 13-acctate (PMA) were obtained from Sigma. 4', 6-diamidino-2-phenylindole (DAPI) was purchased from Beyotime; Pierce®Protein Concentrator PES and 10 K MWCO were purchased from Thermo Fisher Scientific. BCA Protein Assay Kit was purchased from Beyotime. DuoSet ELISA kits was purchased from R&D Systems.

The following antibodies were used: HMGB1 (A2553, Abclonal, USA), Calreticulin (ab2907, abcam, UK) and HSP70 (HSPA1A and HAPA1B could be recognized, ab2787, abcam, UK), GAPDH (10,494–1-AP, Proteintech Technology, USA), Anti-Rabbit Alexa488 (A-11070, Invitrogen, USA), pIRE1α (NB100-2323, Novus, USA), LC3 (Ml52-3, MBL, Japan). The following antibodies were purchased from Cell Signaling Technology (USA): ATF4 (11815S), HSP90 (the total protein of HSP90, HSP90AB1 and HSP90AA1 could be recognized, 4874S), IRE1α (3294S), PARP (9532S), p-PERK (3179S), PERK (5683S), p-eIF2α (9721S), P62 (16177S), RIP(4920S), RIP3 (13526S), XBP-1 s (12782S) and Ki67 (9449S).

#### Cell counting kit-8 assay

Cells were cultured in a 96-well plate with a density of 2000 cells per well, and were incubated with varying drug (S4) concentrations (0.01, 0.1, 1, 10 and 100 μM) for 24, 48, 72 h respectively. CCk-8 was incubated at 37 °C for 1 to 4 h per well. The absorbance value of cells in each well was detected at 450 nm with a multifunctional microplate reader.

#### Colony formation assay

Cells were cultured in 6-well plates at a density of 2000/ well, and treated with 60 μM S4 or 0.05% DMSO. After 2 weeks, cells were fixed with 4% paraformaldehyde and stained with crystal violet for 20 min. The colo.nies were then washed slowly with water and dried at room temperature. The colonies were photographed and counted with Image J software.

#### Spheroid formation and cell death assay

Glioma cells were plated at (1 × 10^3^/well) in ultra-low adhesion 96-well plates, incubating with serum-deprived DMEM/F12 medium containing 20 ng/ml basic FGF, 20 ng/mL of EGF, and a proportion of B27 in medium (1:50 v/v) for 5 days to initial formation of spheriods (over 50 μm in diameter), and then cells were treated with 30 μM, 60 μM S4 or 0.05% DMSO for following 10 days. For cell death assay, spheriods were then stained with 10 μg/ml propidium iodide (PI). Subsequently, the spheriods were observed by fluorescence microscopy. And the dead cells were identified by red fluorescent PI staining. The spheriods were photographed and counted. Number of spheriods and positive staining rate of spheriods were analyzed.

#### Immunofluorescence assay

Glioma cells were seeded on coverslips (NEST, 801,008) for 12 h, then treated with drugs according to the purpose of the experiment. Then cells were fixed in 4% paraformaldehyde (PFA). After the blocking in 2% Bovine Serum Albumin (BSA), cells were then incubated with primary antibody (Calreticulin, 1:75; Ki67, 1:250) for 2 h at room temperature, followed by 30 min incubation with secondary antibodies (Anti-Rabbit Alexa 488, 1:1000 or Anti-Mouse Alexa 488, 1:1000) at room temperature. Nuclei were stained with 1 μg/mL DAPI (C1002, Beyotime, China) in PBS. A laser scanning confocal microscope (Leica TCS SP5II) was applied to monitor the immunofluorescence (IF).

### Protein samples of conditioned media

Cells exposed with S4 or vehicle were pretreated with CQ, Z-VAD-FMK, Necrostain-1, 4u8C, ISRIB, GSK2606414 for 24 h, and then the media of these cells were collected and concentrated to 100 μl using Pierce®Protein Concentrator 2–6 ml/10 K filters according to the manufacturer’s instructions. Protein loading buffer was added proportionally, and the samples were boiled at 100℃for 6 min to prepare protein samples.

### Analysis of cell death by flow cytometry

LN229 and U87MG cells with logarithmic growth phase were plated into a 6-well plate and the cells were treated with different concentrations of S4. Then PBS was used to wash the collected cells. Glioma cells were stained with annexin V-FITC and propidium iodide (PI) according to the to the manufacturer’s instructions (Keygen Biotech/KGA108), and the cells were detected on the flow cytometer within 1 h. Three independent tests were used to examine the fraction of apoptotic cells. Doxorubicin (DOX) was used as a positive control for cell death.

### Immunoblotting

Cells were treated with various agents, collected and processed for immunoblotting analysis as previously described [26]. BCA Protein Assay Kit was used to determine protein concentrations. Protein samples were diluted to a final concentration of 0.5 mg/ml. The diluted protein sample was incubated with 200 μl BCA working buffer at 37℃ for 25 min. The absorbance of A562, or other wavelengths between 540–595 nm, was measured with an enzyme label. To quantify changes, the densitometries of protein bands were determined with a calibrated GS-670 densitometer.

### Co-culture experiments and measurement of cytokine concentration

THP-1 cells were seeded on 12-well cell culture plates (2 × 10^4^ cells per well) and differentiated for 48 h with 20 nM PMA to allow attachment. Then THP-1 cells were treated with conditioned media from glioma cells exposed to S4 (or not) for 24 h. Cell-free supernatants were collected and secreted IL-1α and IL-8 in the media were measured using DuoSet IL-1α and IL-8 ELISA kits (R&D Systems, USA) according to the manufacturer’s instructions [27].

### RNA-sequencing

RNA was extracted from S4-treated and vehicle-treated LN229 cells and RNA-sequencing (RNA-seq) was performed by the Novogene Corporation (Beijing, China). RNA-seq data were analyzed as previously described [28]. RNA-seq data have been deposited at the NCBI Gene Expression Omnibus under the accession number GSE205538.

### Quantitative real time PCR (qRT-PCR)

Total cell RNA was extracted with TRIZOL reagent and cDNA was synthesized by reverse transcription using the Prime Script RT Kit. For qRT-PCR analysis, RNA expression was measured using SYBR Premix Ex Taq kit (TaKaRa, Japan). The relative transcription levels of the genes were calculated using the delta-delta-Ct (ΔΔCT) method and GAPDH was normalized as an endogenous control. Primers are the same as previously described [29].

### In vivo tumor xenograft experiment

A preliminary experiment was conducted to detect the toxicity of S4 in vivo and the appropriate concentration for administration. Nude mice were divided into three groups (four mice in each group) with doses of 0 mg/kg, and 7.5 mg/kg of S4, respectively. Nude mice were treated with S4 and weighed every 3 days. As the preliminary experimental group mice did not show any weight loss in 5 weeks, 1 mg/kg and 5 mg/kg of S4 were used in the following in vivo experiment. LN229 cells (1 × 10^6^) were injected subcutaneously into flanks of female BALB/c nude mice (6 weeks old), which were maintained in animal care facilities without specific pathogens. The mice were randomly divided into three groups (*n* = 5), and were intra-tumoral dosed with S4 (0, 1 mg/kg and 5 mg/kg). Tumor growth was monitored using calipers where two perpendicular tumor diameters were measured every 5 days and tumor volume was calculated according to the formula 0.5 × length × width^2^. After 8 weeks, the tumor-bearing mice were sacrificed with ether anesthesia, and xenografts were excised for follow-up experiments.

### Immunohistochemistry

Xylene and different concentrations of alcohol were used for dewaxing paraffin-embedded tissue sections. The processed sections were blocked with goat serum and incubated with anti-Ki67 antibody (1:200) at 4℃ overnight. DAB detection kit was used for immunoperoxidase staining and hematoxylin was used for staining the nucleus. The tissue sections were sealed with neutral resin after alcohol dehydration treatment.

### Statistical analysis

SPSS 16.0 software was used for statistical data analysis. T-test or one-way ANOVA was used for comparison between groups. 0.05 was considered that the difference was statistically significant. The graphs were drawn using GrapPad Prism 8.0.

## Results

### S4 decreases glioma cell viability

**Fig. 1:**
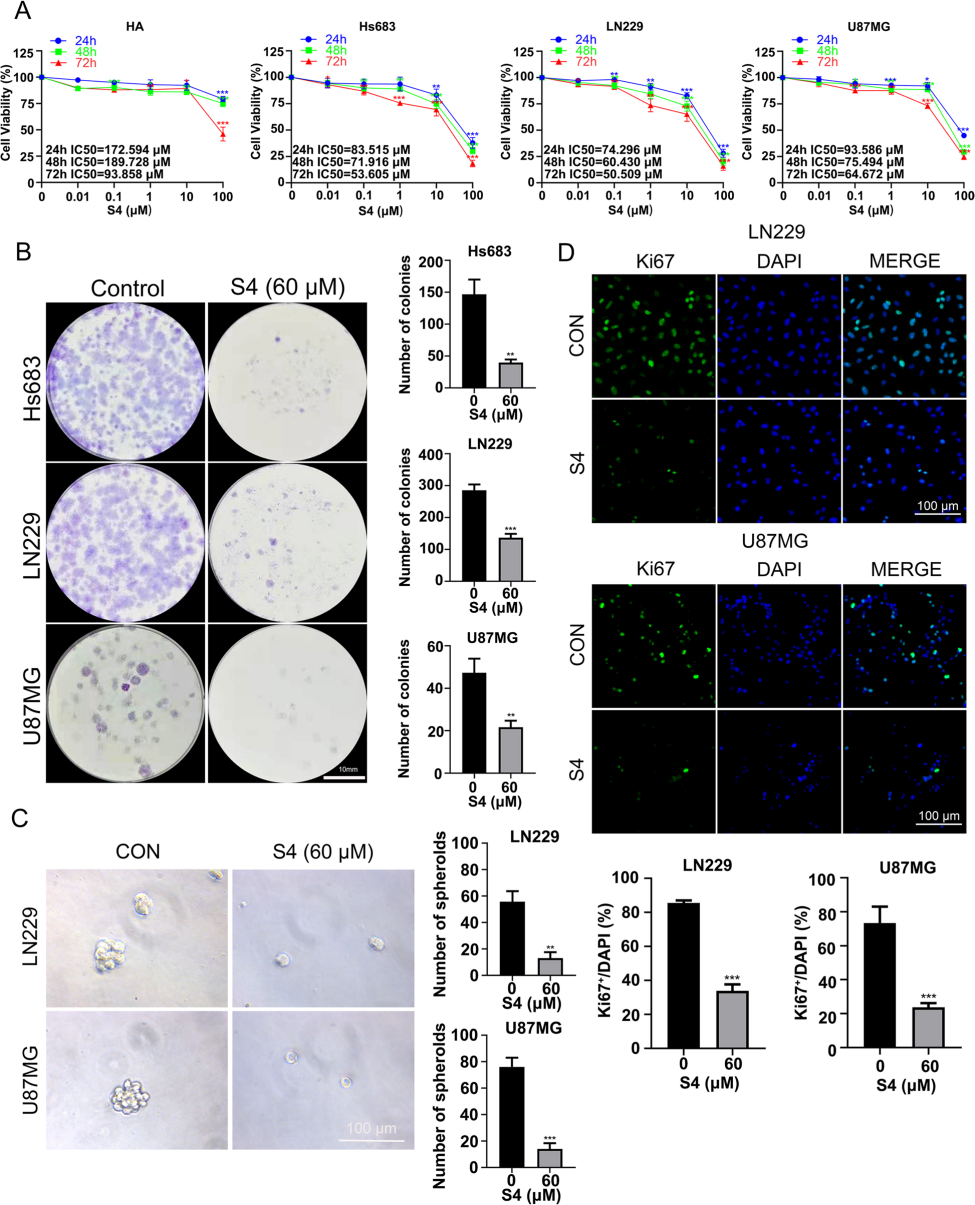
S4 decreases glioma cell viability. **A** HA, Hs683, LN229, and U87MG cells were vehicle-treated or treated with varying concentrations of S4 (0.01,0.1,1, 10, 100 μM) for 24, 48, 72 h respectively. Cell growth was determined using the CCK8 assay. IC 50 values were listed in the margins of each column. **B** Cells (Hs683, LN229, and U87MG) were vehicle-treated or treated with 60 μM S4 and cultured in complete media for 14 days for colony formation analysis. **C** LN229 and U87MG cells cultured in 3D medium were treated with DMSO or 60 μM S4 for 12 days and examined for the spheroid formation. (Scale bar = 100 μm). **D** LN229 and U87MG cells were treated with DMSO or 60 μM S4, then assessed by immunofluorescence staining with ki67 or DAPI. (Scale bar = 100 μm). The above experiments were performed three times (*P < 0.05, **P < 0.01, ***P < 0.001)

We assessed the effect of S4 on the viability of glioma cell lines Hs683, LN229, U87MG and human astrocytes HA cell line by a CCK-8 assay. As shown in Fig. 1A, S4 inhibited the growth of the three glioma cell lines in both dose- and time- dependent manner. This effect was achieved with a much lower half maximal inhibitory concentrations (IC_50_) values for glioma cell lines at each time point than the IC_50_ values for HA cells. Clonogenic growth assays showed that S4 at 60 μM decreased significantly the capability of the glioma cells to grow clonally after a 2-week treatment (Fig. 1B). Three-dimensional (3D) spheroid formation assays indicated that the LN2299 and U87MG microspheroids were substantially lessened in number after 10 days treatment with S4 (Fig. 1C). In addition, 24-h S4 treatment caused a significant decrease in Ki67 staining in both LN229 and U87MG cells (Fig. 1D). Cumulatively, we showed that S4 decreases the viability of glioma cells in vitro.

### S4 induces glioma cell death

**Fig. 2:**
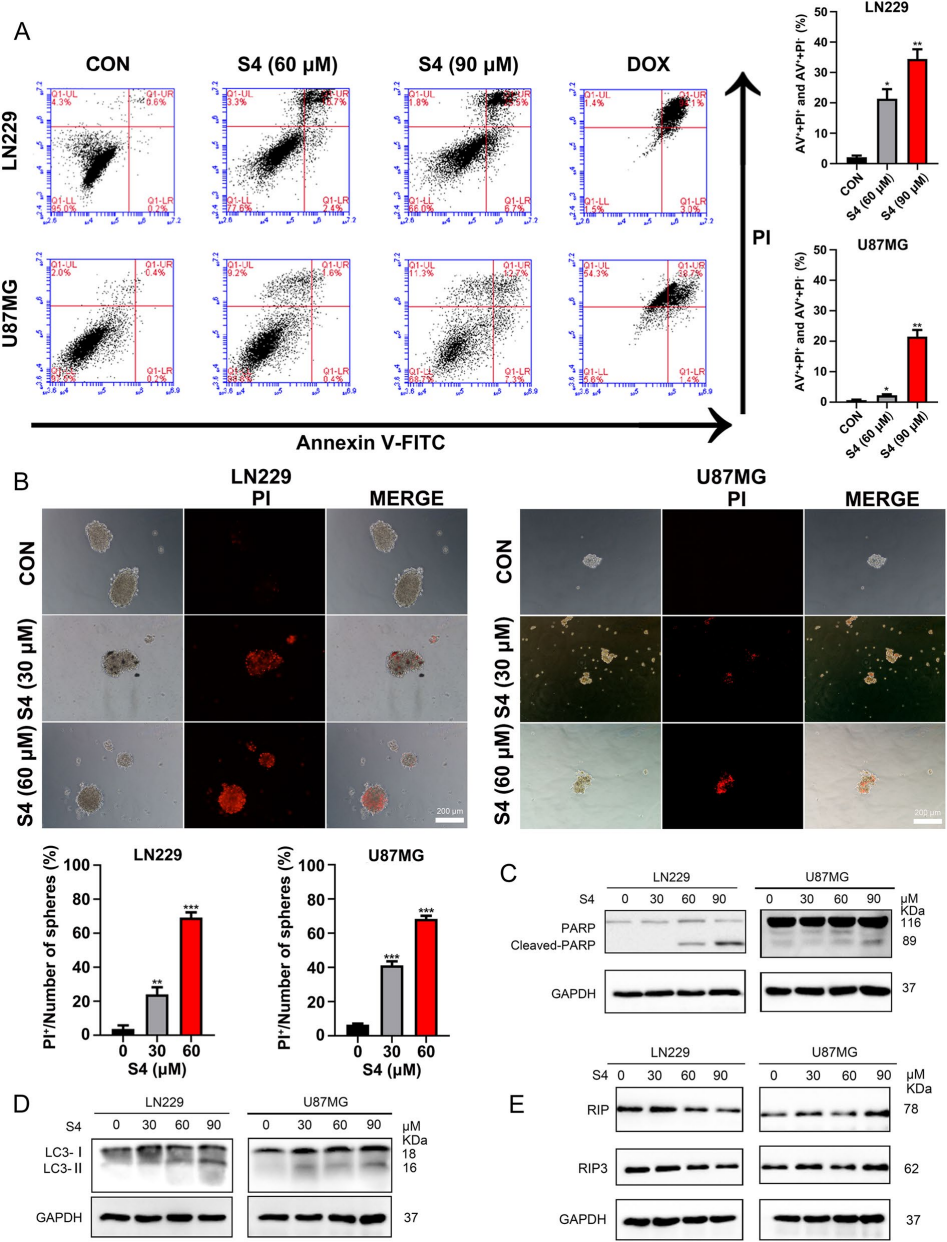
S4 induces glioma cell death. **A** LN229 and U87MG cells were treated with DMSO or S4 (60, 90 μM) for 24 h, stained with annexinV-FITC/PI. Cell death was assayed by flow cytometry. 5 μM concentration of Doxorubicin was taken as a positive control. **B** Spheroids were treated with DMSO or S4(30, 60 μM), stained with PI at 24 h and imaged under phase contrast and red fluorescence microscopy (scale bar = 200 μm). **C**-**E** LN229 and U87MG cells were treated with S4 (0, 30, 60, 90 μM) for 24 h, the relative expression of cleaved-PARP, LC3, RIP and RIP3 was determined by immunoblot analysis. GAPDH was used as a loading control. Experiments were performed three times (*P < 0.05, **P < 0.01, ***P < 0.001)

We next determined whether the suppressed S4-induced growth inhibition in glioma cells was due to cell death. To this end, S4-treated LN229 and U87MG cells were analyzed by flow cytometry with FITC-conjugated Annexin-V and propidium iodide (PI) double staining. As illustrated in Fig. 2A, exposure to S4 at 60 and 90 μM for 24 h significantly increased the percentage of both early and late apoptotic cells in LN229 and U87MG cell lines, suggesting an induction of apoptotic cell death. Doxorubicin (Dox) was used a positive control, which substantially increased the number of apoptotic cells as expected. In addition, we observed large amounts of the cells from S4-treated spheroids derived from glioma cells were stained with the cell-death dye PI, indicative of cell death (Fig. 2B). Furthermore, a dose-dependent cleavage of Poly (ADP-ribose) polymerase (PARP), a classical apoptosis marker, was detectable in S4-treated glioma cells (Fig. 2C). In addition, we also detected an increase in microtubule-associated protein 1 light chain 3 (LC3)-II (an autophagy marker) levels in glioma cells upon exposure to S4, suggesting that S4 might induce autophagy in these cells (Fig. 2D). No obvious change in the levels of RIP1/3, two key proteins involved in necrosis, was detected in S4-treated glioma cells (Fig. 2E).

### S4 induces CRT exposure and release of HMGB1 and HSP70/90 in glioma cells

**Fig. 3:**
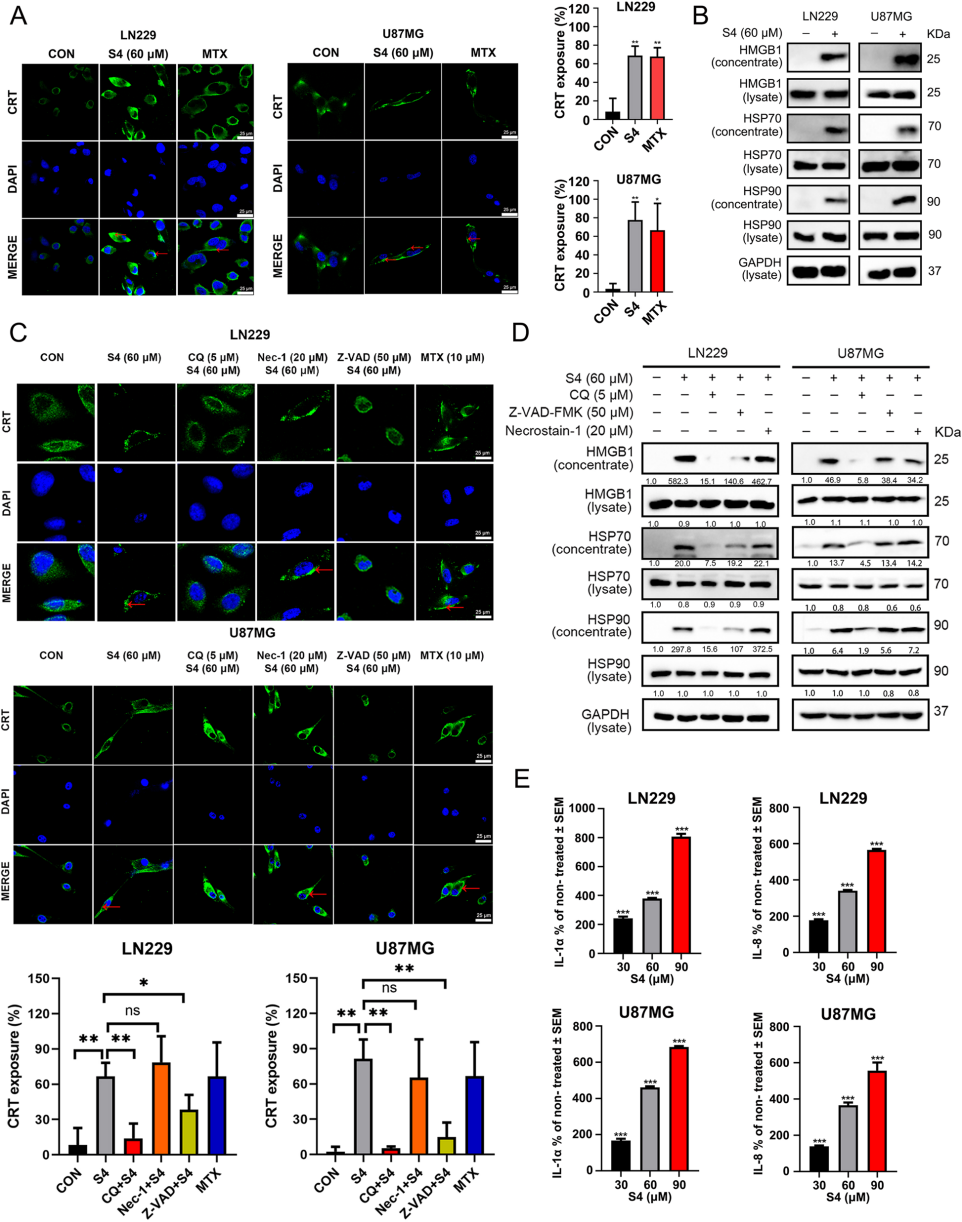
Autophagy and apoptosis are involved in S4-induced immunogenic cell death in glioma cells. **A** LN229 and U87MG cells were treated with DMSO or S4 (60 μM) for 24 h, then stained with an anti-CRT antibody (Green). DAPI was used for nuclear staining (blue). The exposure of calreticulin (CRT) was assessed by confocal imaging. Mitoxantrine (MTX) was used as a positive control. ImageJ software was used to calculate the percentage of CRT positive area (**p < 0.01). Arrowheads indicate positive area. Images are representative of three independent experiments. (scale bar = 25 μm). **B** LN229 and U87MG cells were treated as in (**A**), cell lysates were collected and concentrated. HMGB1 and HSP70/90 expression were measured by immunoblot (IB) analysis. GAPDH was used as a loading control. **C** LN229 and U87MG cells were pre-treated with either Z-VAD-FMK (50 µM), or chloroquine (CQ, 5 µM), or Necrostain-1 (20 µM), following treatment with S4 (60 μM) for 24 h, then exposure of CRT (green) was assessed by immunofluorescence staining. DAPI was used for nuclear staining (blue). MTX was used as a positive control. Arrowheads indicate positive area. (scale bar = 25 μm) (**D**) LN229 and U87MG cells were treated as in (**C**), cell lysates and cell-free supernatants (concentrated) were collected. HMGB1 and HSP70/90 levels were measured by IB analysis. GAPDH was used as a loading control. (**E**) LN229 and U87MG cells were treated with DMSO or S4 (30, 60, 90 μM). Release of IL1α and IL-8 from THP-1 cells co-cultured with conditioned media of S4-treated glioma cells was measured by ELISA. The release rate of control group was 100% for quantitative statistics. The above experiments were performed three times (*P < 0.05, **P < 0.01, ***P < 0.001)

We next investigated whether S4 could trigger ICD in glioma cells by examining the ICD markers including HMGB1and HSP70/90 in cellular supernatants and CRT expression (ecto-CRT) in cell surface. Mitoxantrine (MTX), a known ICD inducer [30], was chosen as a positive control. As illustrated in Fig. 3A, confocal imaging of S4-treated LN229 and U87MG cells revealed a significantly increased exposure of CRT on the cell surface compared with DMSO-treated cells. As expected, MTX treatment induced a strong exposure of CRT in both glioma cell lines. To detect the secreted DAMPs such as HMGB1 and HSP70/90 in S4-treated glioma cells, the cell culture media were collected and concentrated after a 24 h exposure to S4 or DMSO. and the levels of above proteins were assayed by immunoblotting. As depicted in Fig. 3B, a robust increase in protein levels of both HMGB1 and HSP70/90 was detected in conditioned media of S4-treated LN229 and U87MG cells. The above findings indicated that S4 might trigger ICD in glioma cells. Given that the incidence of ICD is generally acknowledged to be tightly connected with programmed cell death such as apoptosis, autophagy and necroptosis [31–33], we then tested whether apoptosis, autophagy and necroptosis would play a role in S4-triggered ICD. To this purpose, we pretreated the cells with an autophagy inhibitor chloroquine (CQ), a necroptosis inhibitor Necrostain-1 (Nec-1), and a pan-caspase inhibitor Z-VAD-FMK (Z-VAD), respectively. The effective concentrations of these inhibitors were selected by a dose–response assay for each compound to prevent cytotoxicity (data not shown). As shown in Fig. 3C, S4-induced translocation of CRT on cell surface in glioma cells was significantly attenuated by pretreatment with either CQ or Z-VAD-FMK, but not Nec-1. Moreover, both CQ and Z-VAD-FMK substantially blocked the release of HMGB1 and HSP70/90 in LN229 and U87MG cells upon exposure to S4, while Nec-1 could not exhibit similar effects (Fig. 3D). To further demonstrate ICD induced by S4, we examined inflammatory cytokine release induced by extracellular HSP70/90 from THP-1 cells. The release of cytokines IL-1α and IL-8 in cell conditioned media from glioma cells exposed to 30, 60 and 90 μM S4 were dramatically increased (Fig. 3E).

### ER stress pathway is involved in S4-mediated immunogenic cell death behavior

**Fig. 4:**
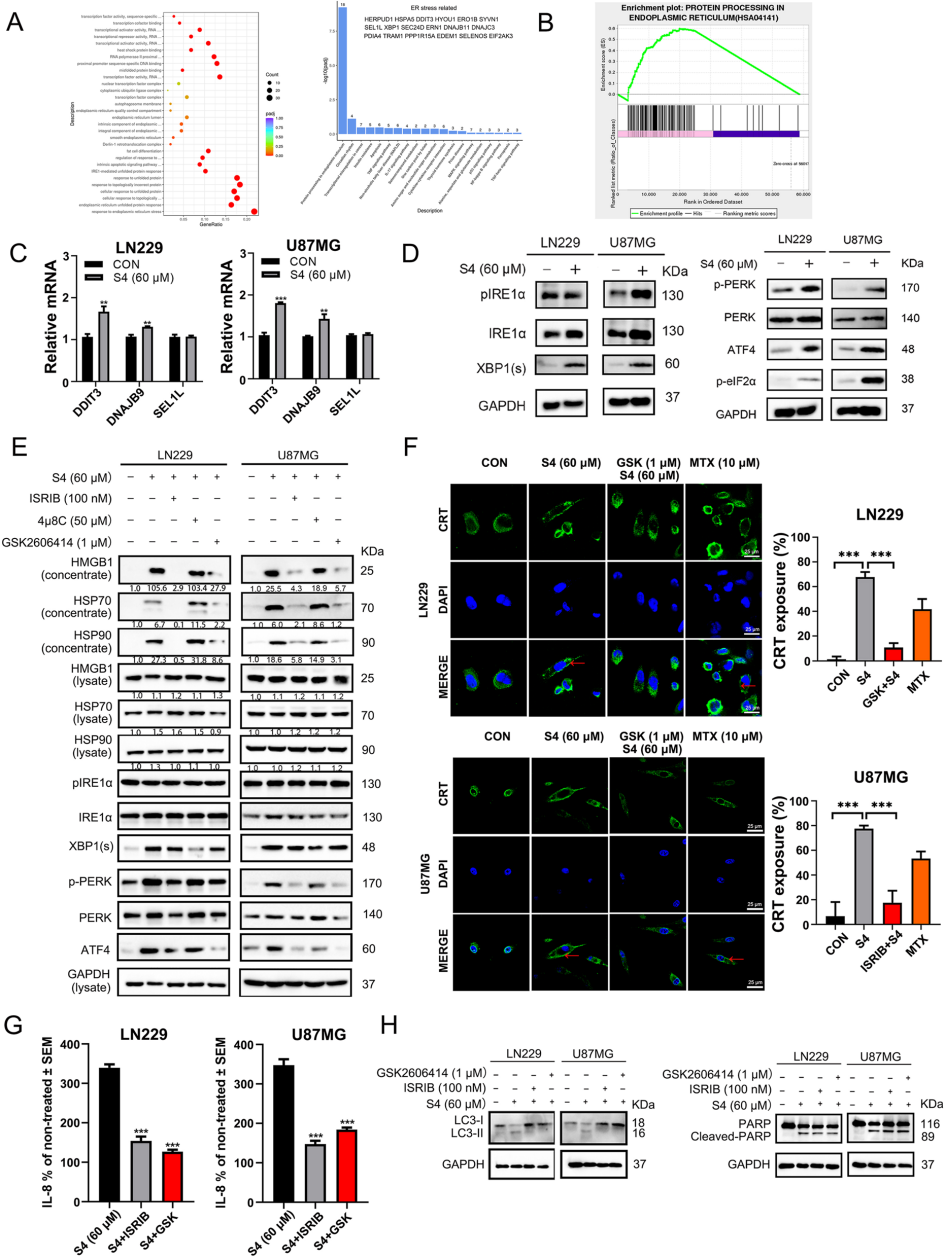
PERK pathway contributes to S4-induced immunogenic cell death in glioma cells. **A** LN229 cells treated with S4 (60 μM) or DMSO for 12 h were collected for transcriptome sequencing. Differentially expressed genes were analyzed by gene ontology. 18 differential genes related to ER stress were also listed. **B** Gene set enrichment analysis of S4-treated LN229 cells and cells treated with vehicle. **C** Real-time quantitative PCR analysis for DDIT3, DNAJB9, and SEL1L mRNA levels of S4-treated LN229 or U87MG cells and control cells. **D** Immunoblot (IB) analysis was performed to detect the action of the PERK pathway related proteins P-perk, ATF4, P-elf2α, and the IRE1α-XBP1 pathway related proteins pIRE1α, IRE1α, XBP1(s). **E** LN229 and U87MG cells were treated either IRE1α -xbp1 pathway inhibitor 4μ8C (50 μM), or PERK pathway inhibitors GSK2606414 (1 μM) and ISRIB (100 nM) with S4 (60 μM) for 24 h and the expression of ER stress related protein, HMGB1 and HSP70/90 was measured by IB analysis. **F** LN229 and U87MG cells were treated with S4 (60 μM) and GSK2606414 or ISRIB respectively, the CRT exposure was detected by confocal microscopy (scale bar = 25 μm). Arrowheads indicate positive area. **G** With or without S4 treatment, LN229 and U87MG cells were pretreated with GSK2606414 or ISRIB, and the media was collected to culture THP-1 cells. Release of IL-8 from THP-1 cells co-cultured with conditioned media was measured by ELISA. The release rate of control group was 100% for quantitative statistics. **H** LN229 and U87MG cells were treated with S4 (60 μM) and GSK2606414 or ISRIB respectively, expression of LC3II and cleaved-PARP was examined by IB analysis. The above experiments were performed three times (*P < 0.05, **P < 0.01, ***P < 0.001)

To explore the signaling pathways involved in S4-mediated ICD behavior in glioma cells, we performed RNA sequencing (RNA-seq) analysis to compare gene expression profiles between S4-treated LN229 cells and cells treated with vehicle. The gene ontology analysis demonstrated that the differentially expressed genes regulated by S4 were largely enriched in ER stress and unfolded protein response (UPR) pathways (Fig. 4A). Moreover, as shown in Fig. 4B, a significant enrichment for gene set was involved in ER stress pathway, suggesting a role for the ER stress pathway in S4-mediated ICD. To validate this, quantitative real-time PCR was used to detect the influence of S4 on mRNA levels of downstream target genes of ER stress signaling pathway. As target genes of three branches, DDIT3 (CHOP) and DNAJB9 (IRE1α-XBP1), but not SEL1L (ATF6) were obviously upregulated in glioma cells after treatment of S4, which declared ATF6 was not the main activated branch (Fig. 4C). We next examined the activation of PRKR-like endoplasmic reticulum kinase (PERK)-eIF2α axis, and inositol-requiring enzyme 1 alpha (IRE1α)-X-box binding protein 1 (XBP1) axis, two major upstream players in ER stress pathways. S4 treatment caused a substantial increase in the levels of XBP1 and the phosphorylated elF2α in both LN229 and U87MG cells (Fig. 4D), confirming an induction of ER stress pathway. To ascertain the role of ER stress pathway in S4-mediaed ICD, glioma cells were pre-incubated with ER stress pathway inhibitors GSK2606414 and ISRIB ( both targeting PERK), and 4μ8C (targeting IRE1α) following S4 treatment. As shown in Fig. 4E, pre-treatment of LN229 and U87MG cells with either GSK2606414 or ISRIB substantially reversed S4-induced secretion and release of HMGB1 and HSP70/90 while 4μ8C failed to do so, suggesting that the PERK-eIF2α axis plays a major role in S4-mediated ICD. Consistently, pre-exposure to GSK2606414 in LN229 cells or ISRIB in U87MG cells significantly blunted the translocation of CRT on cell surface induced by S4 (Fig. 4F). Furthermore, pretreatment of GSK2606414 and ISRIB in S4-exposed LN229 and U87MG cells showed a distinct decrease release of IL8 from THP-1 cells cultured with the conditioned meida (Fig. 4G). In addition, both GSK2606414 and ISRIB markedly reduced S4-induced LC3II expression in either LN229 or U87MG cells (Fig. 4H, upper panels). In addition, S4-iduced cleaved PARP was not affected by either GSK2606414 or ISRIB (Fig. 4I, lower panels). Altogether, these data suggest that these PERK inhibitors might antagonize S4-induced autophagy in the tested glioma cells.

### S4 reduces glioma growth in mice models

**Fig. 5:**
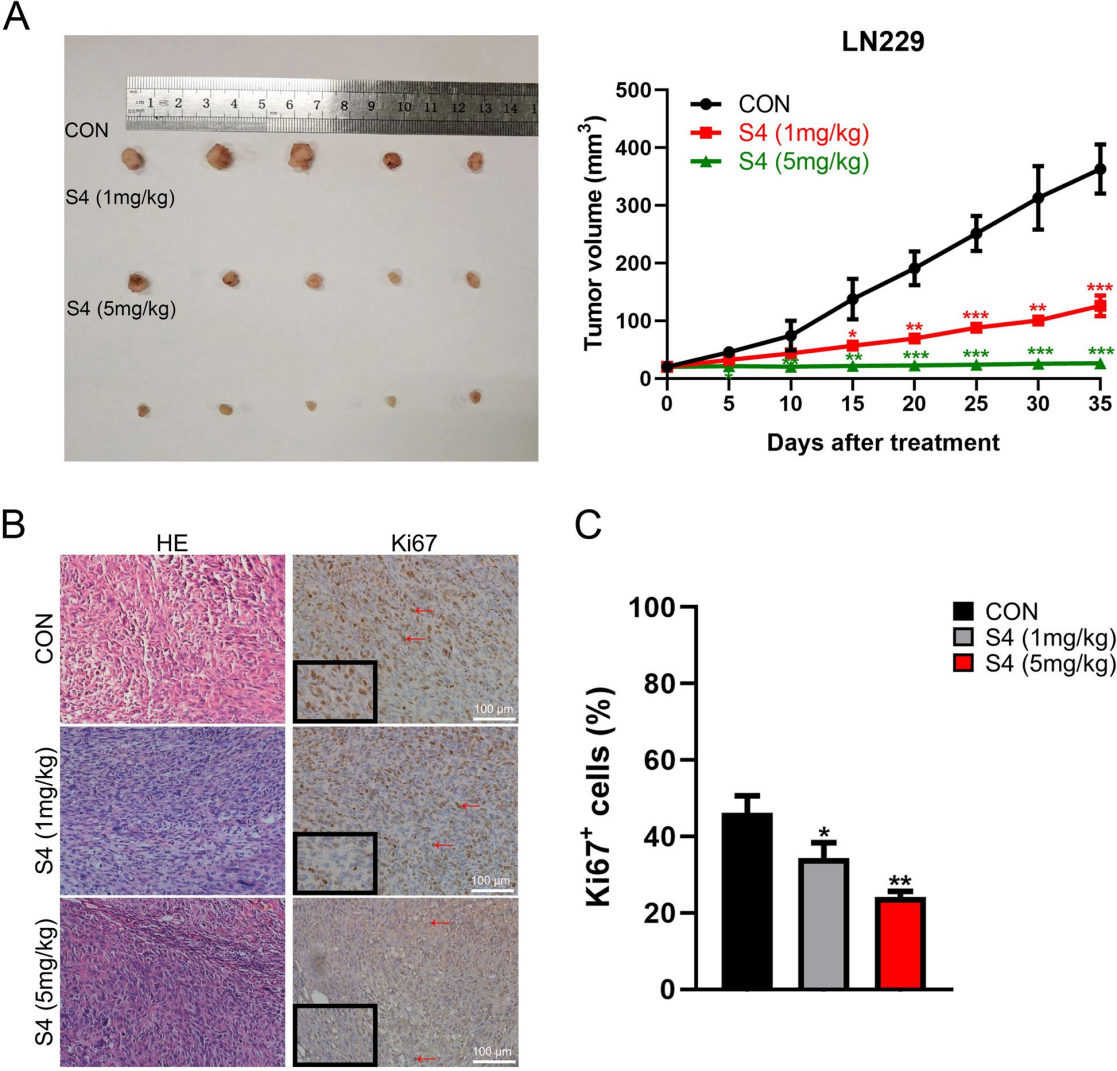
S4 reduces glioma growth in mice models. **A** LN229 cells were injected subcutaneously into the right flanks of mice to establish tumors. When tumors reached approximately 25 mm.^3^, mice received an intratumoral injection of either DMSO, or S4 (1 mg/kg, 5 mg/kg respectively) every three days, *n* = 5 in each group. Tumor volumes were measured at 5-day intervals for 35 days after injections and expressed as the Mean ± SD. Tumor volume-time curves to show any differences in tumor regression. **B** Hematoxylin and eosin (H&E) was used to examine tumor tissues, and immunohistochemistry assay was performed to detect Ki67 expression. Arrowheads indicate positive area (scale bar = 100 μm). **C** IHC analysis of Ki67 expression were performed. (*P < 0.05, **P < 0.01, ***P < 0.001)

To examine the effects of S4 in vivo, mice with LN229-derived tumors were injected with S4 at two different doses (1 mg/kg and 5 mg/kg). As illustrated in Fig. 5A, both dose of S4 significantly reduced tumor growth without notable toxicity, while treatment with 5 mg/kg dose of S4 achieved stronger effects. In addition, Ki67 expression in mice tumor tissue samples in S4-treated group and control group was assessed by immunohistochemistry. Figure 5B and C showed Ki67 staining was evidently weakened in tumor samples of mice treated with S4 at either dose.

## Discussion

In the present study, we provide evidence that the CAIX inhibitor S4 suppresses the growth of glioma cell in vitro and in vivo. Of note, we demonstrate that S4 triggers the expression of ICD markers in glioma cells via the ER stress pathway, indicating that S4 might be a novel ICD inducer. These findings merit further investigation to explore how S4 may be of use in glioma treatment.

The main finding of our study is that the CAIX inhibitor S4 could trigger ICD in glioma cells. ICD has emerged as a key component of therapy-induced anti-tumor immunity. Increasing evidence shows the propensity to undergo ICD as a prognostic factor associated with longer survival in cancer patients in general including glioblastoma patients [34]. Several chemotherapeutic agents such as cyclophosphamide [35], and oxaliplatinum [36], have shown to induce ICD in glioma in vitro and in mouse models [4]. Our data indicates that the CAIX inhibitor S4 might be a novel ICD inducer at least in glioma cells. To understand the underlying mechanism, we performed RNA-seq analysis of differentially expressed genes of cells treated with S4 or mock-treated, and found that the ER stress pathway is robustly enriched among the deregulated signaling pathways. ER stress is mainly accompanied by three sensors: PERK- eIF2α axis, which is pathognomonic for ICD, activating transcription factor 6 (ATF6), and inositol-requiring 1 (IRE1). We observed the elevated eIF2a phosphorylation and increased XBP1 levels, both markers of ER stress, in S4-treated glioma cells. Notably, pharmacological inhibition of the PERK-eIF2α axis reversed S4-triggered induction of ICD markers. It should be pointed out that eIF2alpha phosphorylation is also considered as a hallmark of immunogenic cell death [37]. Therefore our data supports a role of ER stress in S4-induced ICD. ER stress is known to play a pivotal role in eliciting ICD [38–40]. Our recent work showed that ER stress is involved in oncolytic Newcastle disease virus-induced ICD in melanoma cells [41]. Altogether, both our current and previous work further highlight the recognized notion that ER stress plays a major role in intracellular signaling pathways that induce ICD [38]. We further tested how ER stress regulates S4-triggered ICD in glioma cells. Based on our findings that, inhibition of autophagy reduces S4-induced ICD, while inhibition of PERK antagonizes S4-induced autophagy, we could infer that ER stress plays a role in S4-induced ICD at least in part via autophagy. One of the limitations of our work is that how S4 evokes the ER stress in glioma cells remains unknown. And it should be pointed out that the finding that S4 triggers ICD is largely achieved in in vitro experiments, therefore further work is needed to examine the direct influence by S4-mediated ICD on the immune environment in glioma.

Besides, we found that there was no data to support whether S4 could penetrate the blood–brain barrier (BBB). Although characteristics of S4 such as small molecular weight (335.38 g/mol) and fat-soluble provide the theoretical potential for S4 to cross the BBB, we chose subcutaneous model in testing for glioma efficacy at an early stage. S4 could be modified or packaged if necessary for cross-BBB delivery in future research. We hope that our study will be helpful to the broader application of S4.

## Conclusions

In conclusion, we show the CAIX inhibitor S4 as a novel ICD inducer in glioma cells. Our findings highlight a novel mechanism for the antitumor actions of the CAIX inhibitor S4 and warrant further investigation of S4-induced ICD in clinical application.

## Supplementary Information

**Additional file 1.**

## Data Availability

All data reported in this paper will be shared by the lead contact upon request.
