# Supplementary material for: Carbonic anhydrase IX inhibitor S4 triggers release of DAMPs related to immunogenic cell death in glioma cells via endoplasmic reticulum stress pathway
Source: Cell Commun Signal. 2023 Jun 29;21:167. doi: 10.1186/s12964-023-01180-7 (PMC10311836; doi:10.1186/s12964-023-01180-7)

WB Data

Fig.2 C

LN229 PARP/Cleaved PARP

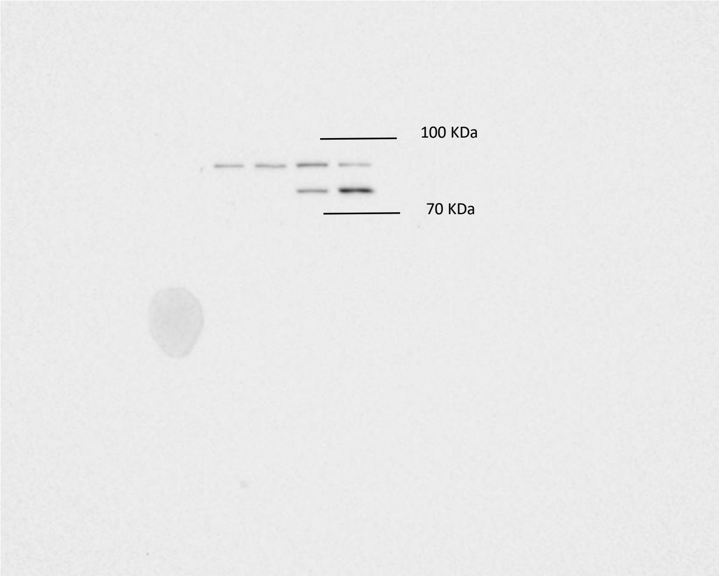

U87MG PARP/Cleaved PARP

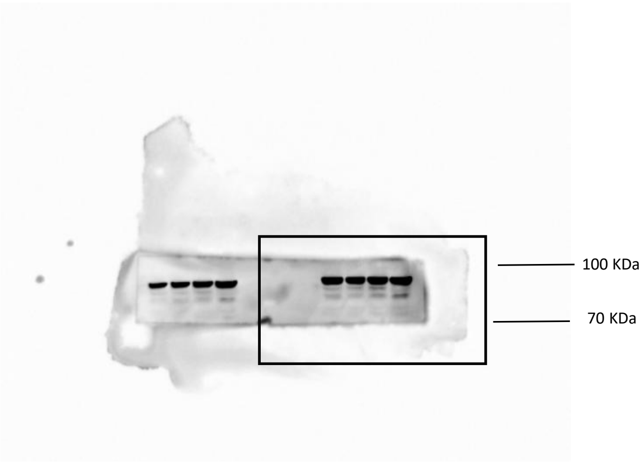

LN229 GAPDH

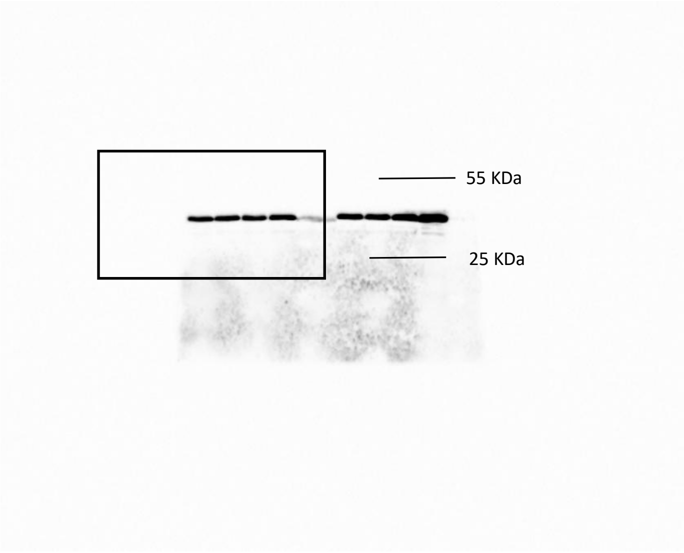

U87MG GAPDH

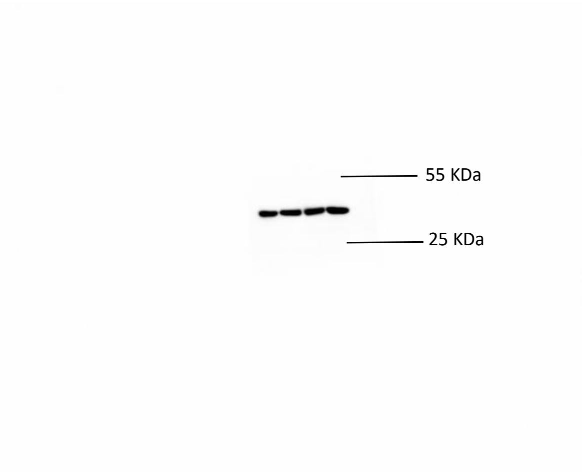

Fig.2 D

LN229 LC3

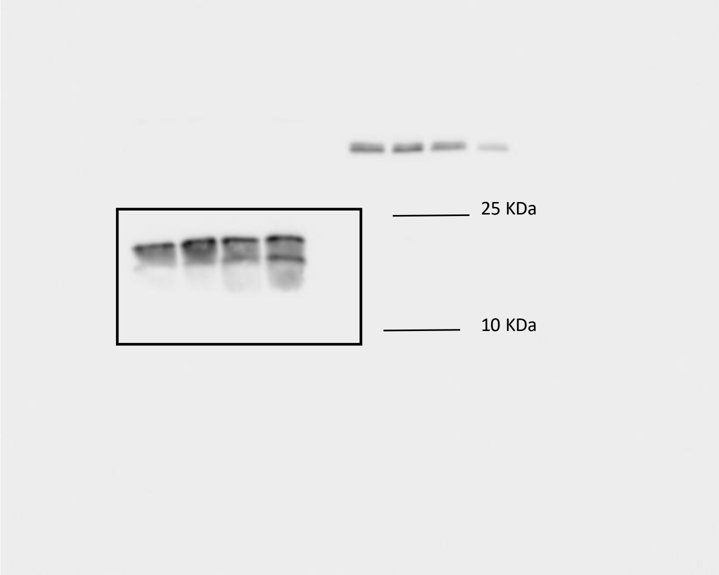

U87MG LC3

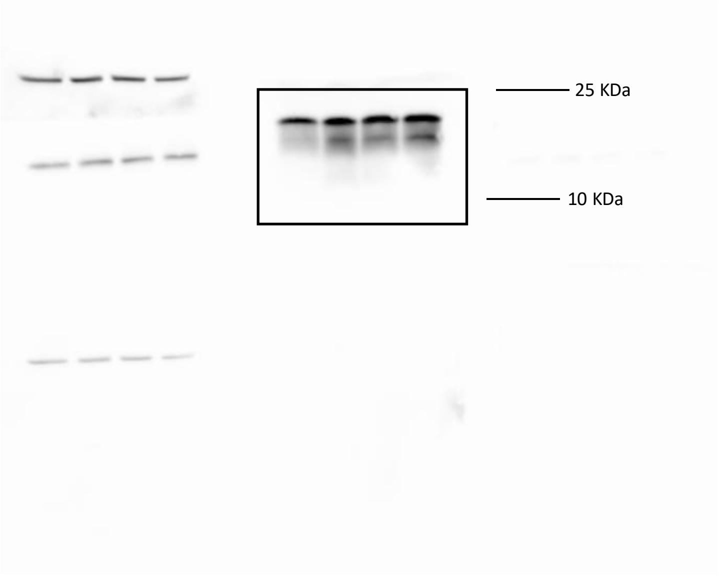

LN229 GAPDH

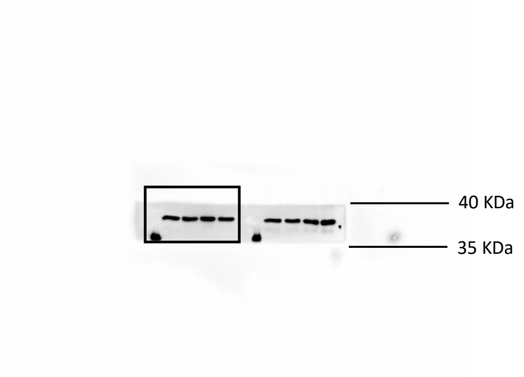

U87MG GAPDH

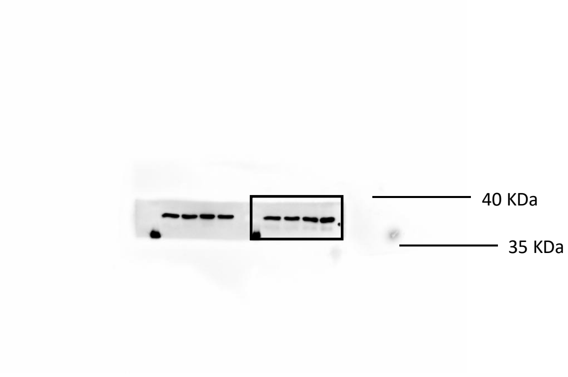

Fig.2 E

LN229 RIP

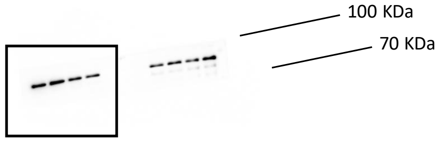

U87MG RIP

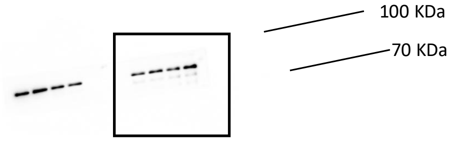

LN229 RIP3

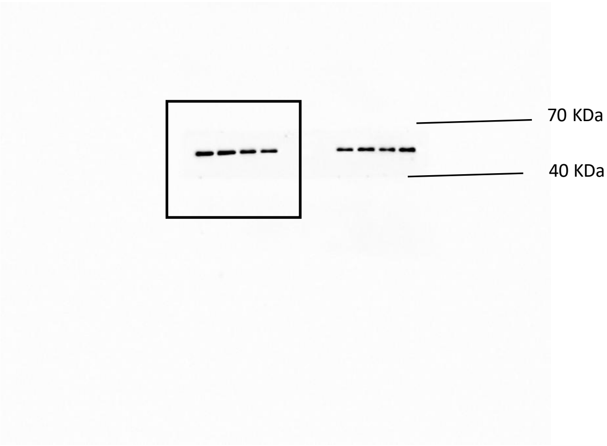

U87MG RIP3

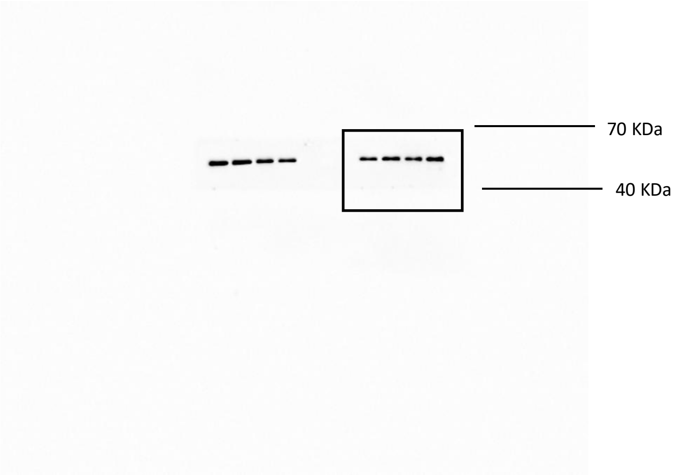

LN229 GAPDH

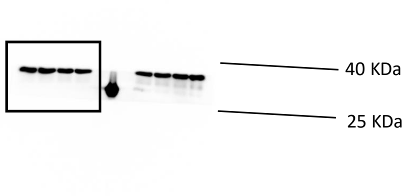

U87MG GAPDH

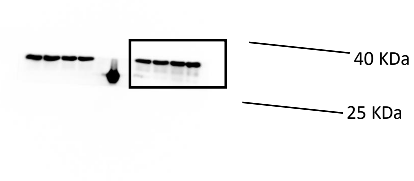

Fig.3 B

LN229 HMGB1(concentrate)

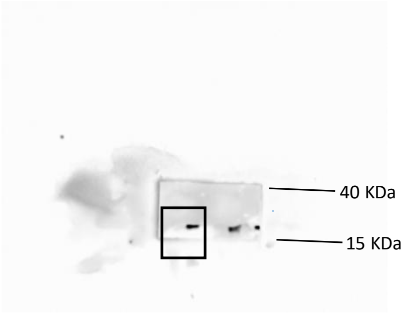

LN229 HMGB1(lysate)

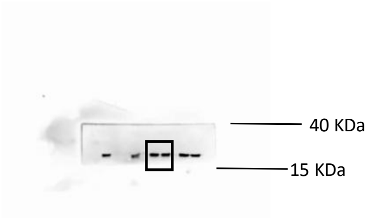

LN229 HSP70(concentrate)

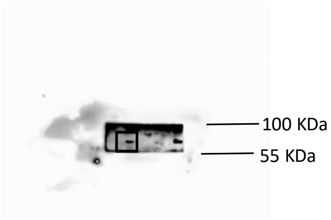

LN229 HSP70(lysate)

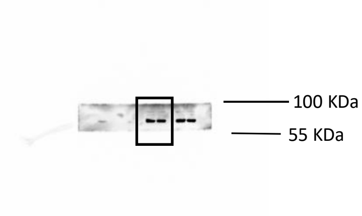

LN229 HSP90(concentrate)

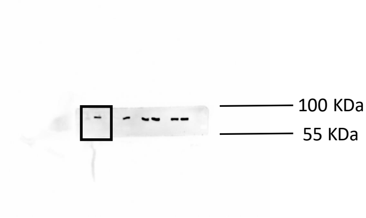

LN229 HSP90(lysate)

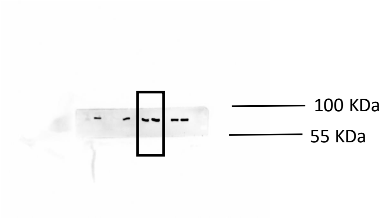

LN229 GAPDH(lysate)

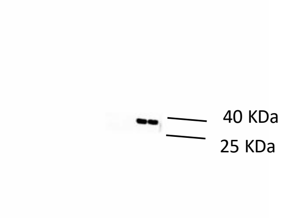

U87MG HMGB1(concentrate)

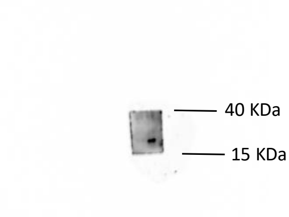

U87MG HMGB1(lysate)

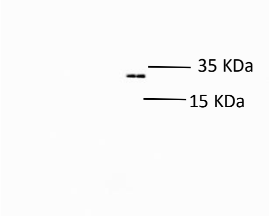

U87MG HSP70(concentrate)

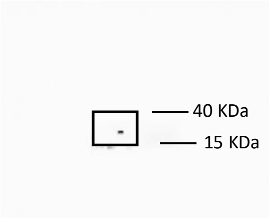

U87MG HSP70(lysate)

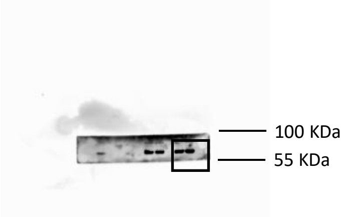

U87MG HSP90(concentrate)

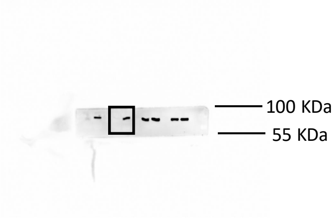

U87MG HSP90(lysate)

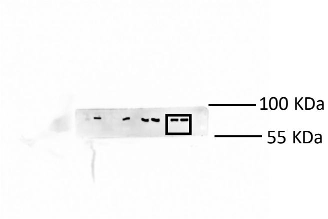

U87MG GAPDH(lysate)

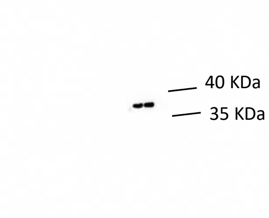

Fig.3 D

LN229 HMGB1(concentrate)

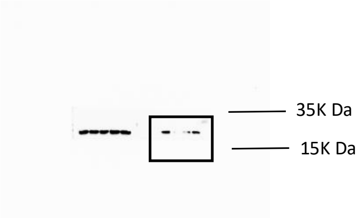

LN229 HMGB1(lysate)

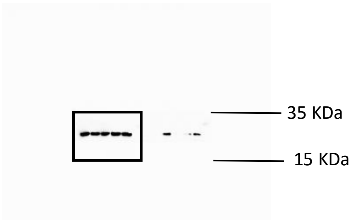

LN229 HSP70(concentrate)

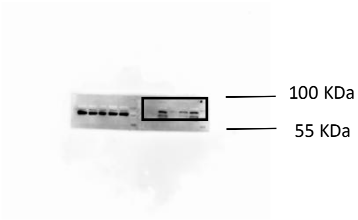

LN229 HSP70(lysate)

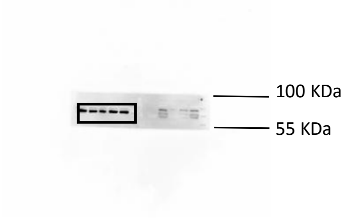

LN229 HSP90(concentrate)

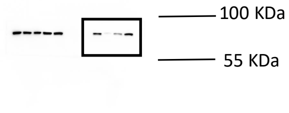

LN229 HSP90(lysate)

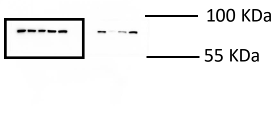

LN229 GAPDH(lysate)

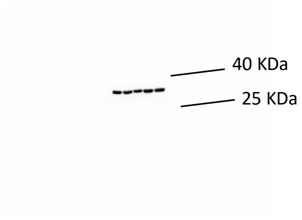

U87MG HMGB1(concentrate)

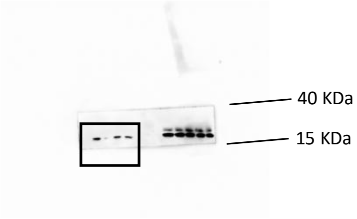

U87MG HMGB1(lysate)

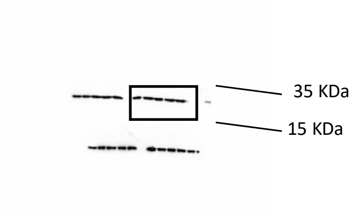

U87MG HSP70(concentrate)

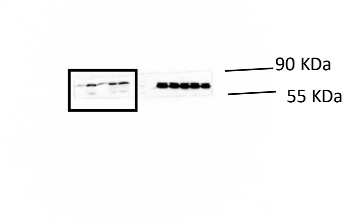

U87MG HSP70(lysate)

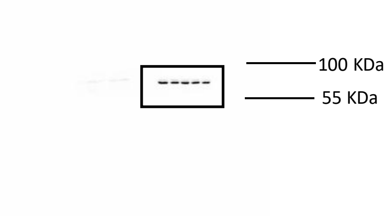

U87MG HSP90(concentrate)

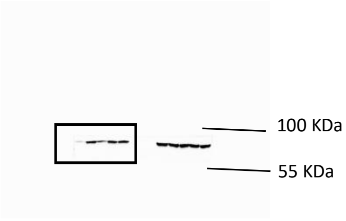

U87MG HSP90(lysate)

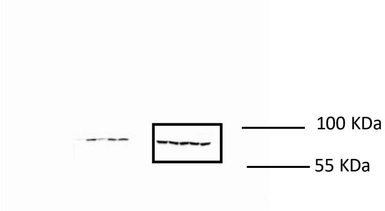

U87MG GAPDH(lysate)

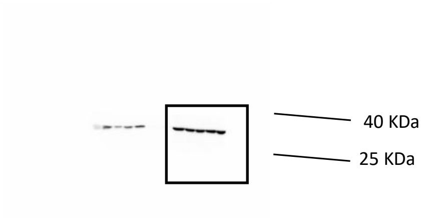

Fig.4 D

LN229 pIRE1 α

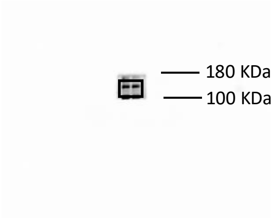

LN229 IRE1 α

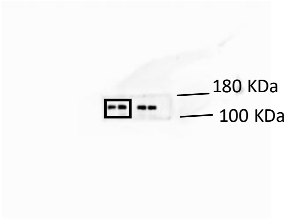

LN229 XBP1(s)

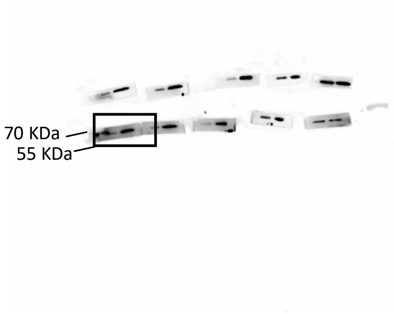

LN229 GAPDH

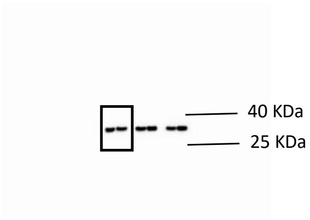

U87MG pIRE1 α

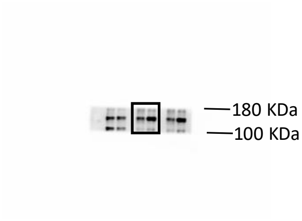

U87MG IRE1 α

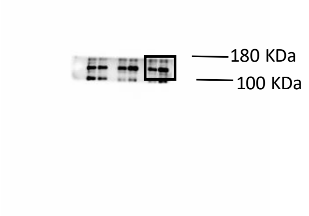

U87MG XBP1(s)

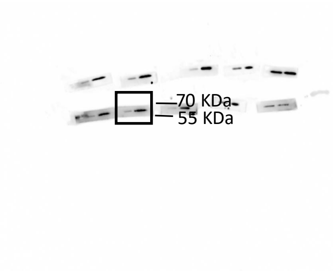

U87MG GAPDH

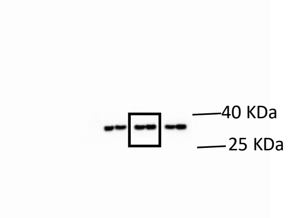

LN229 PERK

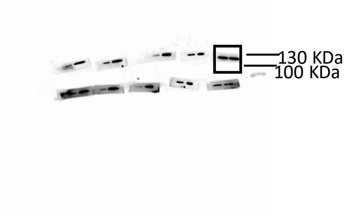

LN229 p-PERK

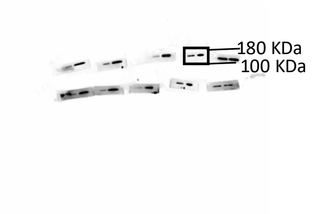

LN229 ATF4

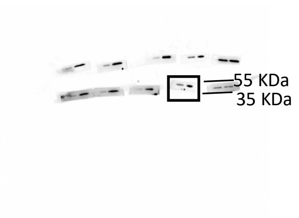

LN229 p-eIF2 α

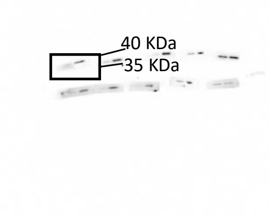

LN229 GAPDH

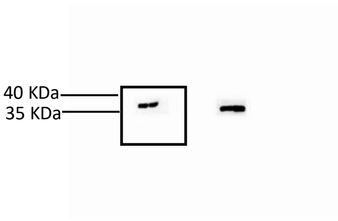

U87MG PERK

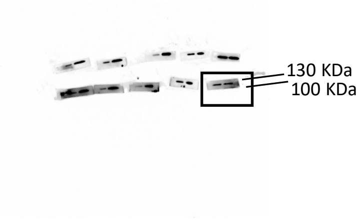

U87MG p-PERK

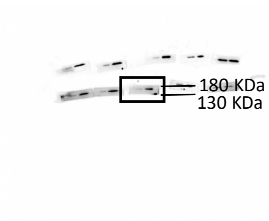

U87MG ATF4

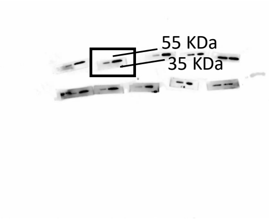

U87MG p-eIF2 α

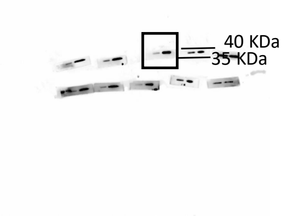

U87MG GAPDH

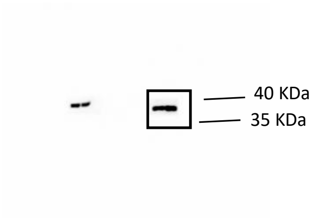

Fig.4 E

LN229 HMGB1(concentrate)

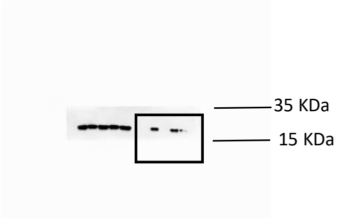

LN229 HMGB1(lysate)

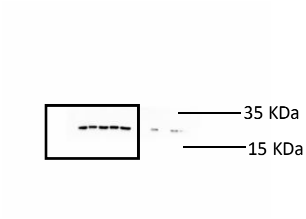

LN229 HSP70(concentrate)

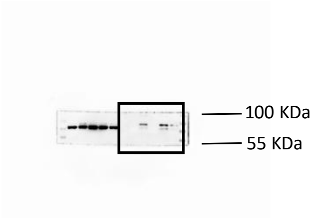

LN229 HSP70(lysate)

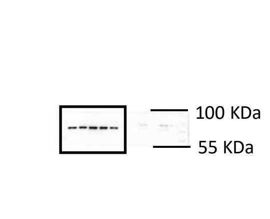

LN229 HSP90(concentrate)

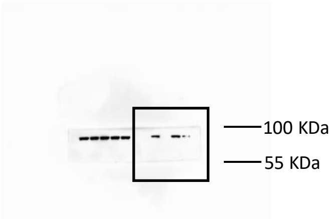

LN229 HSP90(lysate)

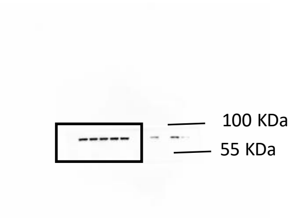

U87MG HMGB1(concentrate)

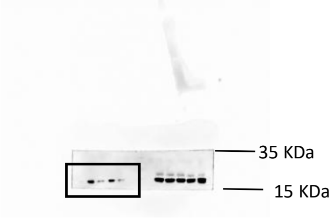

U87MG HMGB1(lysate)

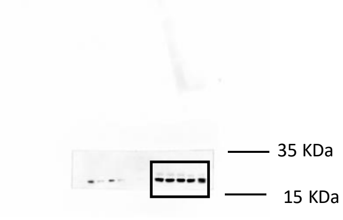

U87MG HSP70(concentrate)

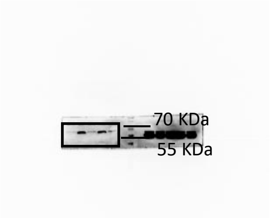

U87MG HSP70(lysate)

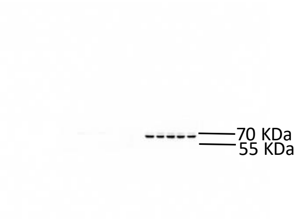

U87MG HSP90(concentrate)

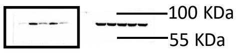

U87MG HSP90(lysate)

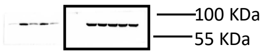

LN229 p-IRE1 α

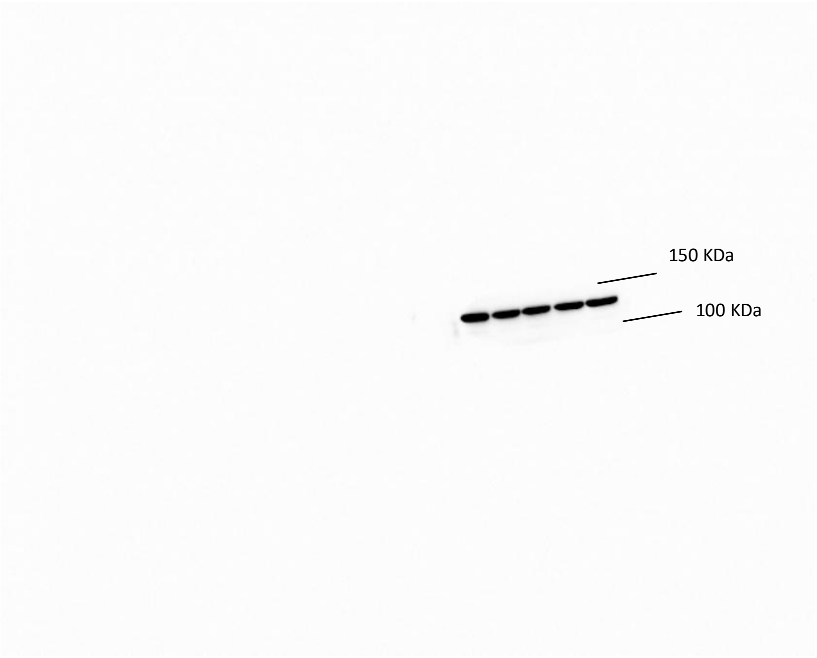

U87MG p- IRE1 α

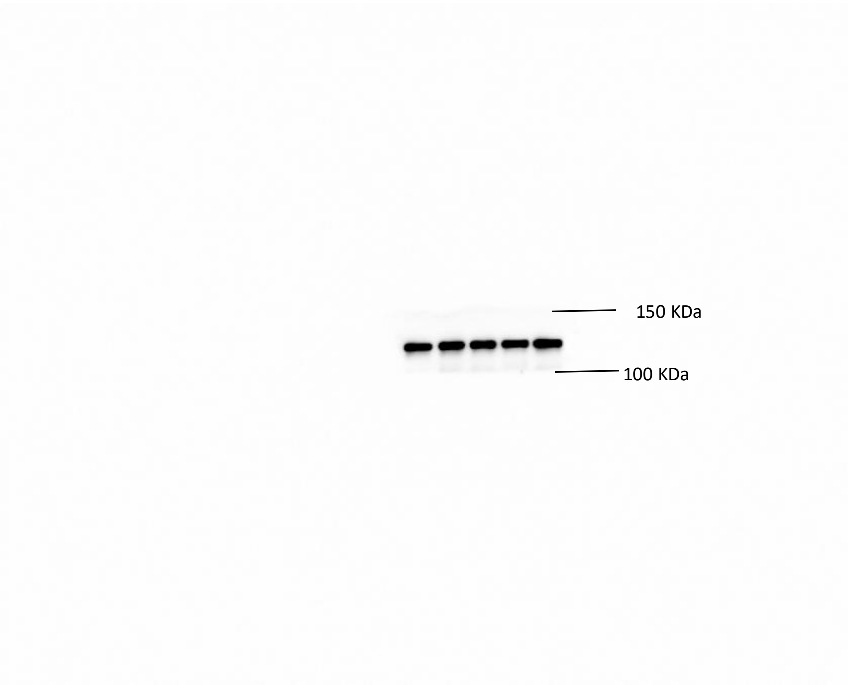

LN229 IRE1 α

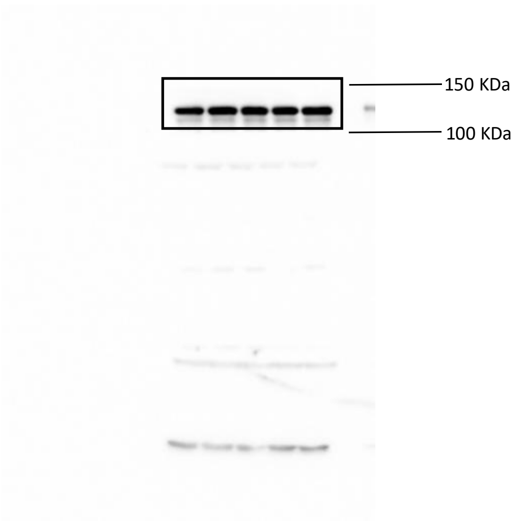

U87MG IRE1 α

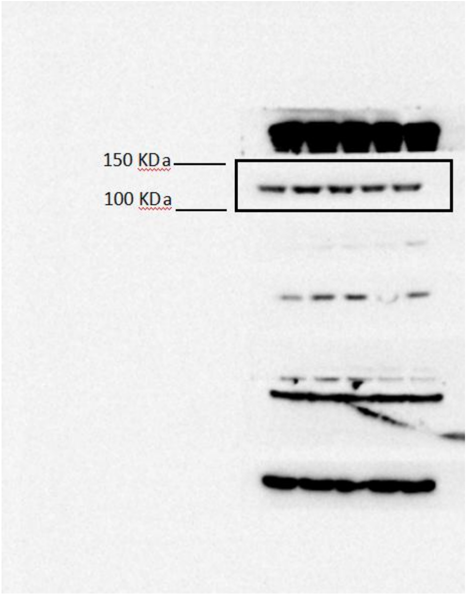

LN229 XBP1(s)

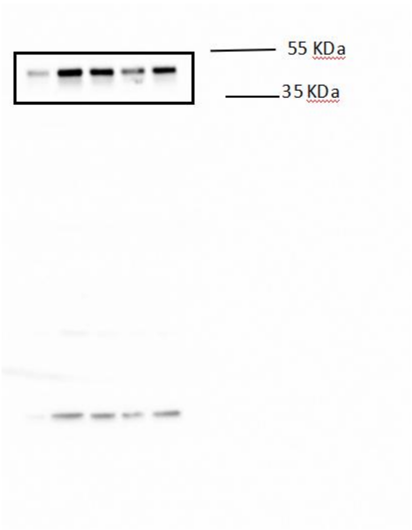

U87MG XBP1(s)

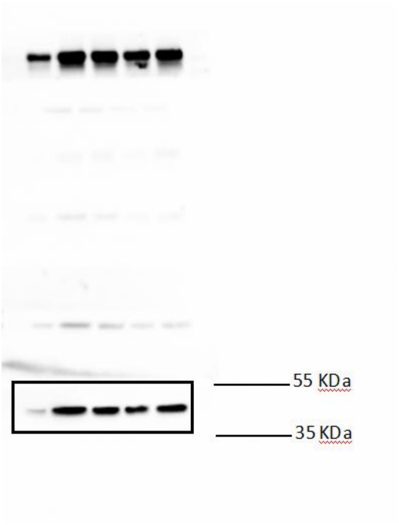

LN229 p-PERK

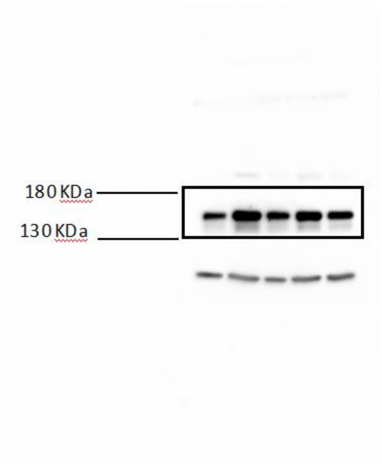

U87MG p-PERK

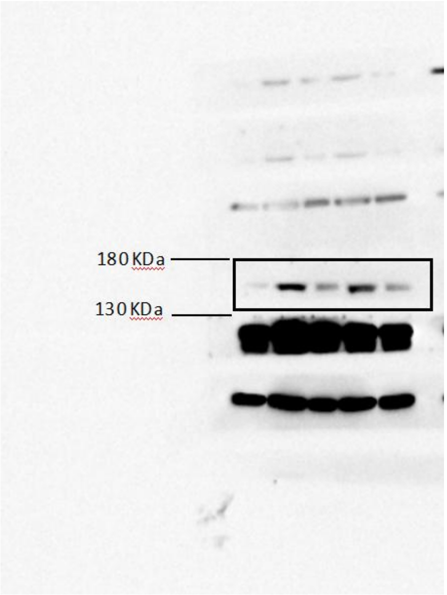

LN229 PERK

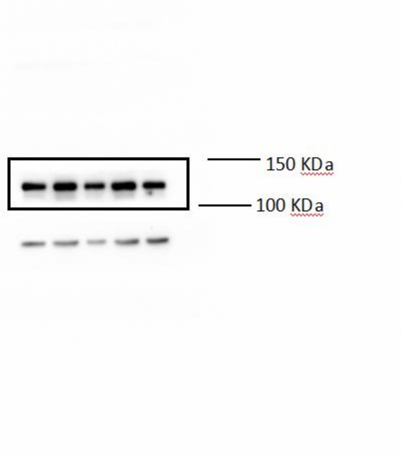

U87MG PERK

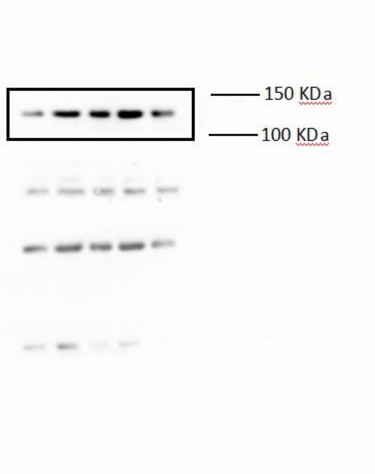

LN229 ATF4

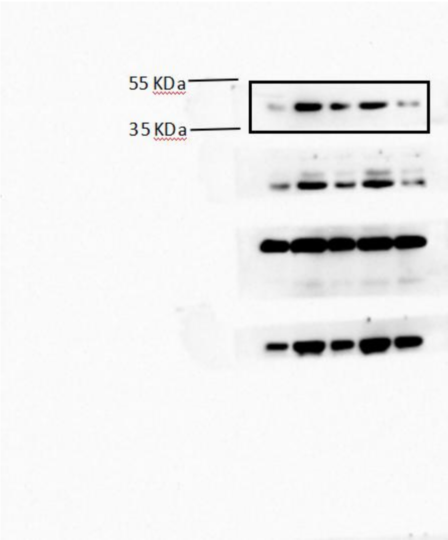

U87MG ATF4

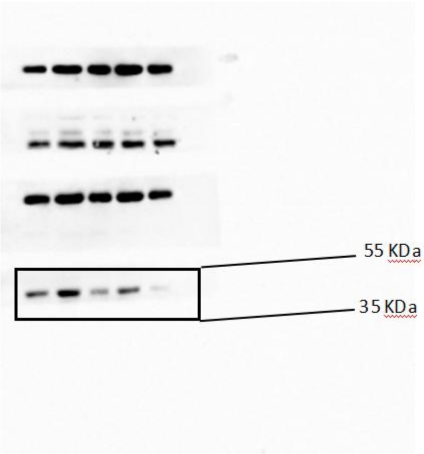

LN229 GAPDH(lysate)

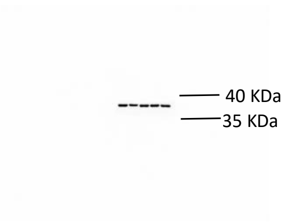

U87MG GAPDH(lysate)

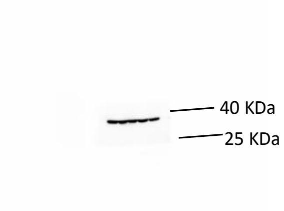

Fig.4 H

LN229 LC3

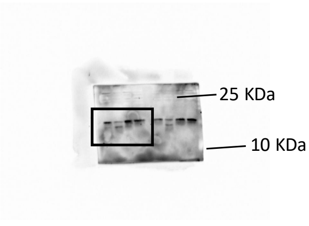

LN229 GAPDH(LC3)

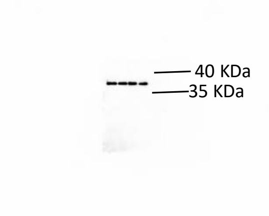

U87MG LC3

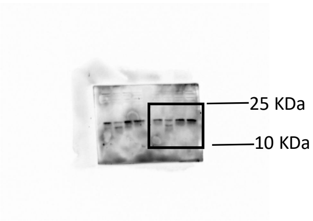

U87MG GAPDH(LC3)

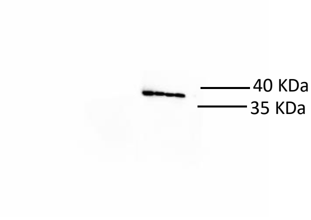

LN229 PARP/Cleaved-PARP

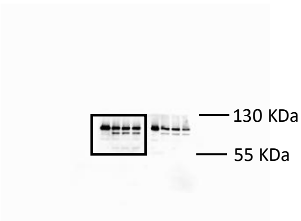

LN229 GAPDH(PARP)

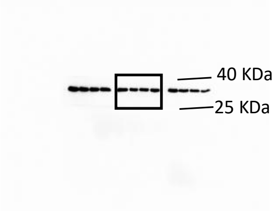

U87MG PARP/Cleaved-PARP

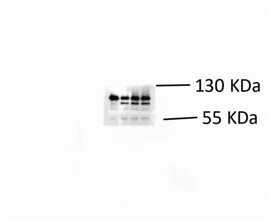

U87MG GAPDH(PARP)

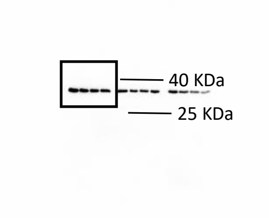

Supplement: Supplementary file 2 — Additional file 1. [file 12964_2023_1180_MOESM1_ESM.pdf]
